# A retrospective analysis of risk factors of oromaxillofacial infection in patients presenting to a hospital emergency ward

**DOI:** 10.1186/s40902-019-0238-9

**Published:** 2019-11-22

**Authors:** Jinyoung Park, Jae-Yeol Lee, Dae-Seok Hwang, Yong-Deok Kim, Sang-Hun Shin, Uk-Kyu Kim, Jae-Min Song

**Affiliations:** 1 Department of Oral and Maxillofacial Surgery, School of Dentistry, Pusan National University, Beomeo, Mulgeum, Yangsan, 50612 Republic of Korea; 2 Biomedical Research Institute, Pusan National University Hospital, Busan, South Korea

**Keywords:** Dental infection, Maxillofacial space infection, Odontogenic infection, Risk factor, Hospital admission

## Abstract

**Background:**

The purpose of this study was to review the clinical features of oromaxillofacial infections in patients presenting to a hospital emergency ward, to identify the key factors affecting the requirement for hospitalization, and the potential risk factors predisposing to a prolonged length of hospital stay.

**Methods:**

A retrospective medical record review of the 598 patients treated for oromaxillofacial infection from 2013 to 2017 at the oral and maxillofacial surgery department, Yangsan Pusan National University Hospital, was conducted. The following information was collected from each patient: sex, age, past medical history, site of infection, etiology, admission or outpatient care, level of C-reactive protein (mg/dL), fascial spaces involved, treatment method, and duration of hospitalization. Chi-squared tests were used to identify risk factors, which were further analyzed using multivariable logistic regression.

**Results:**

A total of 606 patients were eligible for inclusion in the study, of which eight were excluded due to having incomplete charts; thus, 598 patients were included: 55% were male, mean patient age was 47.1 ± 19.9 years, and 12.9% of patients were diabetic. Furthermore, 71.2% of patients had infection originating in the mandible; the most common tooth of origin was lower posterior, and 29.8% of patients were hospitalized. Risk factors for hospital admission were elderly patients with concurrent disease, elevated C-reactive protein level, and multiple-space infection in the oromaxillofacial area. The duration of hospitalization was correlated with both diabetes and age.

**Conclusions:**

The requirement for hospital admission is determined by the severity of the infection; even severe infections, once treated with appropriate surgery, have no relation to the length of hospital stay. The important risk factors for increased duration of hospitalization are diabetes mellitus and older age. The understanding of risk factors associated with a prolonged hospital stay during the treatment of oromaxillofacial infection will aid in treatment planning as well as highlight the importance of adequate diabetes control in patients at risk of such infection.

## Background

Oromaxillofacial infections usually occur in the fascial planes and potential spaces of the maxillofacial region due to dental caries, periodontitis, trauma, or endodontic infections [1]. Not knowing that they could be life-threatening if left untreated, prior symptoms and warning signs are often overlooked and it comes to the point where emergency care is inevitable. Due to their complex anatomical characteristics of fascial spaces that are connected to each other, life-threatening complications such as necrotizing fasciitis, respiratory obstruction, descending mediastinitis, brain abscess, and sepsis could occur [2]. Therefore, clinicians should be well aware of the clinical features of oromaxillofacial infections and carefully examine to choose the best treatment regimen in order to decrease the associated mortality rate. It is also crucial to identify the risk factors of these infections to determine the severity of the infection [1].

Through various studies, potential risk factors of oromaxillofacial infections have been identified [3–11]. There are many studies on the necessity of admission treatment [6, 11], the incidence of complications [3], and the relationship with the duration of treatment for the severity of infection due to age [12, 13], diabetes mellitus [4, 14], the number of infected space and infection site [4]. Most studies have been limited to odontogenic infection only. In this study other causes of visits to the dental department such as jaw fracture, cysts, and osteomyelitis infections were also included, in addition to odontogenic infection.

The purpose of this study was to investigate the clinical characteristics of patients with oromaxillofacial infections and to identify the key factors determining the requirement for hospitalization and the factors associated with the length of hospital stay.

## Methods

We reviewed the medical records of patients presenting to the emergency department of Yangsan Pusan National University Hospital, South Korea, and treated for oromaxillofacial infection from 2013 to 2017 at the oral and maxillofacial surgery department of the same hospital. Eight patients with incomplete medical records were excluded, resulting in 598 patients being enrolled in the study. All patients were diagnosed based on the clinical findings and treated using an identical protocol. Patients were defined as those with symptoms of inflammation of the maxillofacial region involving the maxilla, mandible, and teeth.

Information regarding sex, age, past medical history, site of infection, etiology, whether the patient was receiving admission or outpatient care, level of C-reactive protein (mg/dL), fascial spaces involved, complications, treatment method, and duration of hospitalization was collected. Based on this, patients were divided into two groups: hospitalized patients and outpatients. The hospitalized patients were further divided into two groups: those who were admitted for ≥ 12 days and those who were admitted for < 12 days. Based on the collected information, statistical analysis of significant features among the groups was performed.

A database was constructed using Microsoft Excel (Microsoft, Redmond, WA, USA) and imported into SPSS (SPSS Inc., Chicago, USA) for statistical analysis. Descriptive statistics were computed for all variables. Univariable analysis was undertaken to identify the associations between different variables and the patient receiving admission care. Odds ratios and *p* values (based on the chi-square test) were calculated. A *p* value of < 0.05 was considered statistically significant. Significant risk factors were further analyzed using multivariable logistic regression analysis. Descriptive statistics such as frequency and percentage were used to analyze the related factors.

## Results

**Fig. 1:**
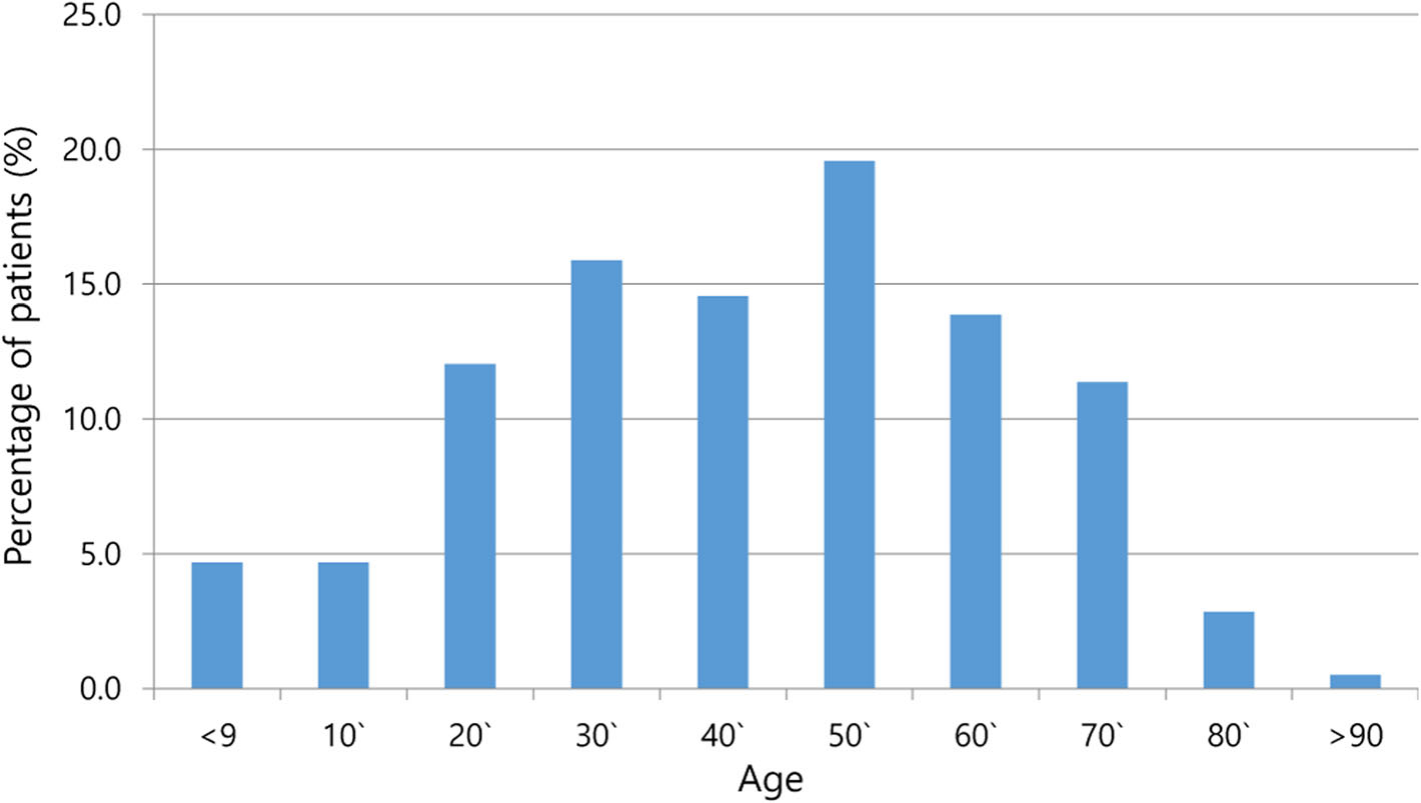
Distribution of patients according to age (%). The age distribution of 598 patients showing the age by decade most commonly affected by infection was 50s, followed by 30s. The number of bar refers to the percentage of patients

Five hundred ninety-eight patients with complete records were included in the study. Eight patients with incomplete records were excluded. The demographic and clinical characteristics of the patients in the study are summarized in Table 1. The mean age of the study subjects was 47.13 ± 19.9 years. The age distribution of the study subjects is shown in Fig. 1. Of the 598 patients, 12.9% were diabetic, while 34.1% had other systemic illness such as hypertension or renal disease. Odontogenic infection in our study originated from a pulpal focus in 493 (82.4%) patients; a further 60 odontogenic infections originated in the extraction socket and sites of dental surgery. In 45 patients (7.53%) the origin was non-odontogenic such as jaw fracture, cystic lesions, and osteomyelitis.

**Table 1:** Basic characteristics of the oromaxillofacial infection patients in this study (*N* = 598)

Variable	Categories	Number of patients	Percentage (%)
Gender	Male	331	55.35
	Female	267	44.65
Age (years)	< 65	468	78.26
	≥ 65	128	21.40
Diabetes	Present	77	12.88
	Absent	521	87.12
Concurrent illness	Present	204	34.11
	Absent	394	65.89
Site of infection	Maxilla	172	28.76
	Mandible	426	71.24
Etiology	Odontogenic	553	92.47
	Non-odontogenic	45	7.53
Admission	Inpatient	178	29.77
	Outpatient	420	70.23
CRP (mg/dL)	≤ 10	460	76.92
	> 10	138	23.08
Involved spaces	Single	528	88.29
	Multiple	70	11.71
Complications	Present	9	1.51
	Absent	589	98.49
Duration of treatment (days)	≤ 6	208	34.78
	> 6	247	41.30
	Loss of follow-up	143	23.91
Involved spaces	Vestibule space	269	44.98
	Primary space	259	43.31
	Secondary space	51	8.53
	Tertiary space	19	3.18
Treatment	Intraoral I & D	353	59.03
	Extraoral I & D	145	24.25
	Medication	70	11.71
	Extraction only	22	3.68
	*etc.	8	1.34

*Includes curettage and cyst enucleation

**Fig. 2:**
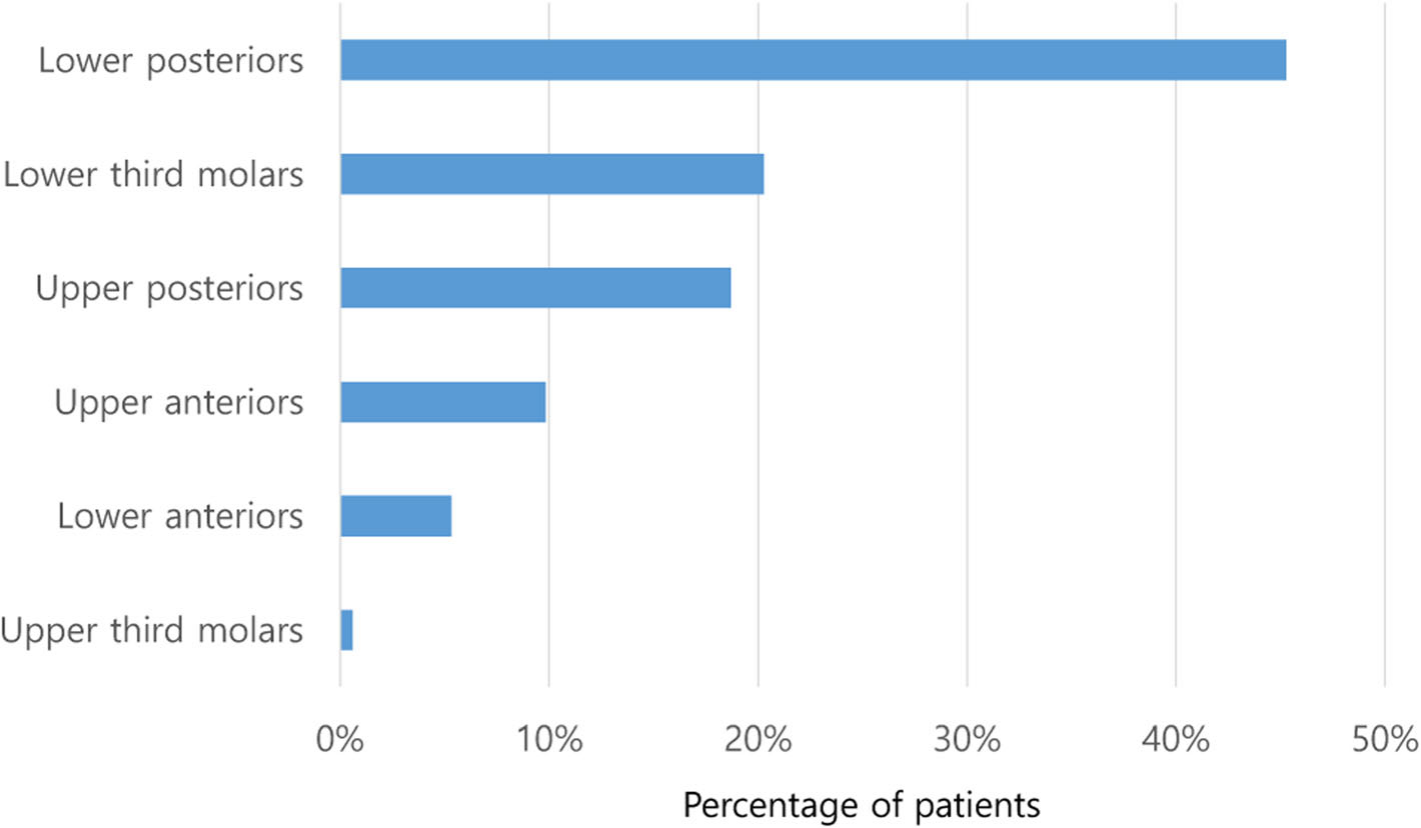
Teeth involved in maxillofacial space infection. The most frequently involved teeth were the lower posteriors, followed the by lower third molars. They are arranged in order of the most frequently involved teeth

**Fig. 3:**
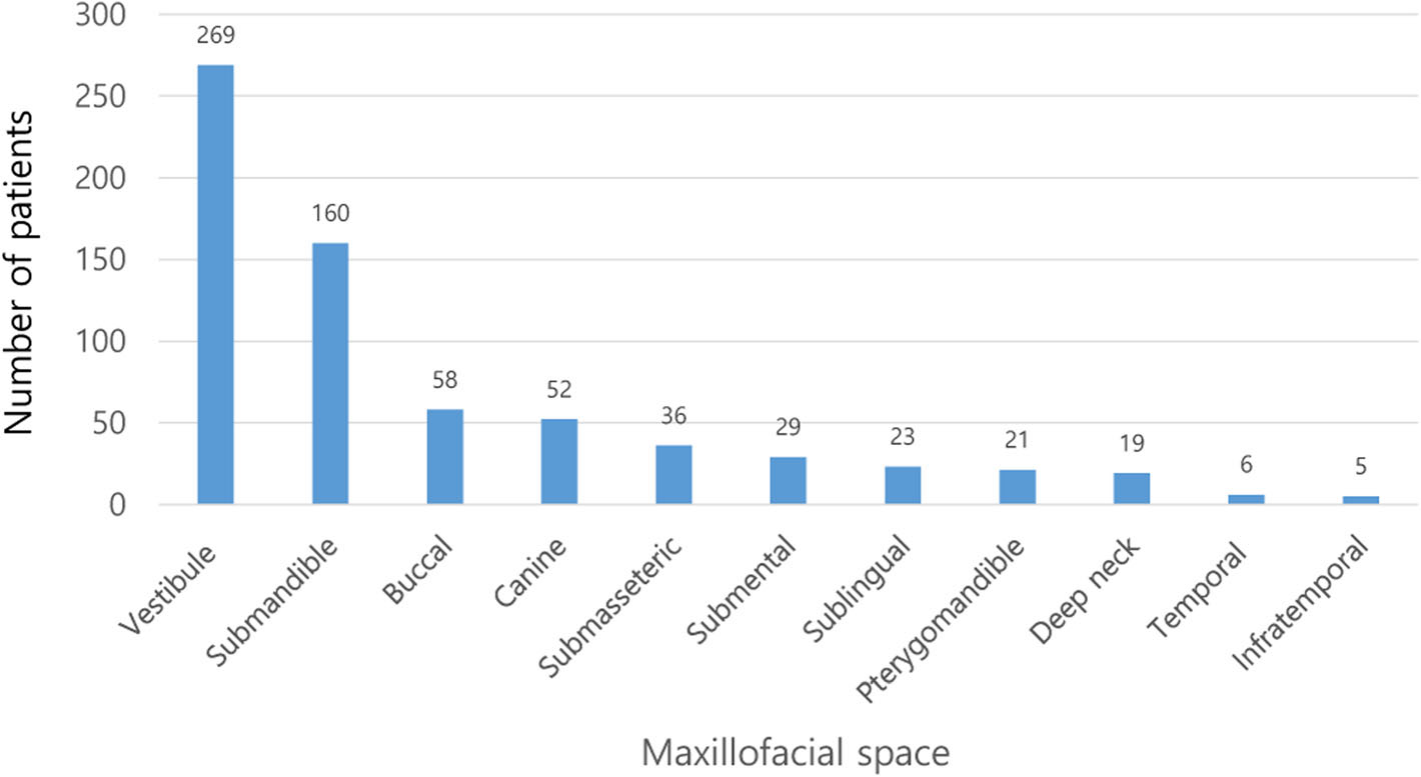
Maxillofacial spaces involved in oromaxillofacial infections. In this study, the most frequently infected area was the vestibular space, followed by submandibular space infection. The number above the graph is the number of infected spaces. If multiple infected spaces were present in one patient, they were counted as duplicates

Of the 598 cases of oromaxillofacial infection, 426 (71.2%) originated in the mandible and 172 (28.8%) originated in the maxilla. The most frequently involved site was the lower posteriors, followed by the lower third molars and upper posteriors (Fig. 2). The most common space involved was the vestibular space (45.0%), followed by the submandibular space (26.8%). The frequency of involvement of different spaces is shown in Fig. 3. Seventy patients (11.7%) had multiple spaces affected.

In total, 178 patients (29.8%) were admitted for management, while 419 patients (70.0%) were managed as outpatients. One patient died in the emergency room due to sepsis. Of the 598 patients, 353 had intra-oral drainage, 145 had extra-oral drainage, 70 had medication, and 22 patients were treated with extraction only. The remaining 8 patients had curettage and cyst enucleation. All patients were prescribed broad-spectrum antibiotics. The mean duration of dressing for outpatients was 7.58 days, and the average length of stay for hospitalized patients was 12.43 days.

The characteristics of the 178 hospitalized patients are detailed in Table 2. It was determined that, compared with patients treated as outpatients, admitted patients were older (*p* = 0.001), and were more likely to have other underlying diseases (*p* = 0.007), a higher level of C-reactive protein (≥ 10 mg/dL) (*p* < 0.001), and multiple-space involvement (*p* < 0.001). Additionally, univariable analysis (chi-square test) identified that the etiology of infection was more likely to be non-odontogenic (*p* = 0.004) and the most common site of infection was the mandible (*p* < 0.001). Multivariable logistic regression analysis showed an association with multi-space involvement (*p* = 0.024), a higher level of C-reactive protein (*p* = 0.003), mandible origin (*p* < 0.001), and non-odontogenic infection (*p* < 0.001) (Table 3).

**Table 2:** Patient characteristics and admission: univariable analysis (*n* = 178)

Variable	Categories	Number of patients	Odds ratio	95% confidence interval	*p* value
Gender	Male	107	1.319	0.924–1.883	0.127
	Female	71			
Age group	< 65	125	0.513	0.341–0.770	*0.001*
	≥ 65	53			
Diabetes	Present	32	1.826	1.117–2.987	*0.015*
	Absent	146			
Concurrent illness	Present	75	1.643	1.143–2.361	*0.007*
	Absent	103			
Etiology	Odontogenic	156	0.411	0.223–0.758	*0.004*
	Non-odontogenic	22			
Site of infection	Maxilla	25			
	Mandible	153	3.295	2.064–5.262	*< 0.001*
CRP (mg/dl)	≤ 10	83	0.100	0.065–0.153	*< 0.001*
	> 10	95			
Involved spaces	Single	117	0.042	0.020–0.087	*< 0.001*
	Multiple	61			

Significant *p* values in italics

**Table 3:** Patient characteristics and admission: multivariable analysis

Variable	*p* value	Odds ratio	95% confidence interval
Odontogenic/non-odontogenic	*< 0.001*	13.245	6.085–28.832
Maxilla/mandible	*< 0.001*	6.092	3.780–9.817
Level of CRP (> 10 mg/dL/≤ 10 mg/dL)	*0.003*	2.273	1.319–3.917
Multiple-space infection	*0.024*	2.311	1.119–4.772

Significant *p* values in italics

Additionally, we analyzed the risk factors of the 178 patients who were hospitalized for 12 or more days. Univariable analysis showed statistical significance in patients of older age (≥ 65 years, *p* = 0.042), diabetes mellitus (*p* = 0.001), a non-odontogenic cause of infection (*p* = 0.004), CRP > 10 mg/dL (*p* = 0.038), and multiple-space infection (*p* = 0.049) (Table 4). The results of the linear regression analysis showed a statistically significant association with age (*p* = 0.001) and diabetes (*p* < 0.001) (Table 5).

**Table 4:** Admitted patient characteristics and longer admission days—univariable analysis

Variable	Categories	Length of hospital stay		Admitted patients	Odds ratio	*p* value	95% confidence interval
		≥ 12 days	< 12 days				
Gender	Male	80	27	107	1.006	0.986	0.505–2.006
	Female	35	36	71			
Age (years)	< 65	88	37	125	2.365	*0.042*	1.017–5.503
	≥ 65	45	8	53			
Diabetes	Present	31	1	32	0.075	*0.001*	0.010–0.565
	Absent	102	44	146			
Concurrent illness	Present	58	17	75	0.785	0.493	0.393–1.570
	Absent	75	28	103			
Etiology	Odontogenic	111	45	156	0.835	*0.004*	0.774–0.900
	Non-odontogenic	22	0	22			
Site of infection	Maxilla	16	9	25			
	Mandible	117	36	153	1.828	0.183	0.745–4.488
CRP (mg/dL)	≤ 10	56	27	83	2.063	*0.038*	1.036–4.106
	> 10	77	18	95			
Involved spaces	Single	82	35	117	2.177	*0.049*	0.993–4.772
	Multiple	51	10	61			

Significant *p* values in italics

**Table 5:** Admitted patient characteristics and longer admission days: multivariable analysis (linear regression analysis)

	Regression coefficient	*p* value
Age (older than 65)	5.450	*0.001*
Diabetes	6.912	*< 0.001*

Significant *p* values in italics

## Discussion

In this study, we retrospectively evaluated a large patient population with oromaxillofacial infections. To our knowledge, there is no retrospective or prospective study available, which evaluates the perspective of orofacial infections in South Korea. The demographic data of our study were consistent with the other studies published in English. In this study, males predominated (55.35%); Zhang et al. [1] reported the proportion of males to be 59.0%. Many other authors also reported a predominance of males, as high as 66% [15, 16]. The mean age of patients in this study was 47.13 ± 19.9 years, similar to that of Zhang et al. [1] (47.5 years) and Allareddy et al. [17] (40 years). Mandibular molars were the most frequently involved teeth in odontogenic infections, with these teeth being the cause of infection in 72.4% of cases in Gholami’s study [11]. Odontogenic infection was implicated in 63% of cases of maxillofacial region infection [12], lower than the 92.47% of the present study. This is because that the patients were referred after classification in an emergency department. In the Ottaviani et al. [2] study, which included the vestibular space, multiple-space infection was reported in 8.86% [2] of cases, compared with 11.71% of our study. In addition, only 2.76% received inpatient treatment, which differed from our result of 29.77%. Excluding the vestibular space, the submandibular is the most commonly infected space, a finding similar to other published studies [13, 16].

Flynn et al. [18] found that severity scoring for the number of infected spaces and the site of the infected space appeared to be valid measures of the severity of infection. In our study, severity of infection such as number of infected spaces [3, 11, 18] and site of infection [4] was associated with the requirement for hospitalization. Sharma et al. [19] found that the level of C-reactive protein (CRP) can be an effective marker for determining the severity of infection, a finding confirmed by other studies [20, 21].

Regression analysis showed a statistically significant association between long-term hospital admission and patients being of an older age and having diabetes. This finding was consistent with other studies [4–6, 12, 15, 22, 23]. Interestingly, several variables indicating the severity of infection were not associated with an increased length of hospitalization. These results do not support the findings of Flynn et al. [18]. Among the papers related to the hospitalization period, the identified risk factors were derived to be irrelevant in this study such as medically compromising diseases (with the exception of diabetes), number of infected spaces [11], and site of infection [18]. This is because older patients and patients with diabetes had lower defense against pathogenic infections, and their recovery rate was low [4]. Host immune mechanisms are important to resolve infection [14]. From those results, regardless of the factors associated with severity of infection, patients can be expected to heal well by removing the infection source and performing proper drainage. If the initial treatment is done properly, length of stay may not be associated with severity of infection.

In this study, a long hospital stay is defined as ≥ 12 days, which was the average length of hospitalization. Patients generally remain hospitalized until the infection resolves or is controlled, and until the patient is returned to a pre-infection state of health. In various studies, the criteria for prolonged hospitalization differ between studies. Usually, hospitalization over the average period is considered long-term admission. In the USA, the average length of stay was 3 to 8.3 days [5, 16, 17, 24]; in Iran, it was 6.8 days [11]; in Finland, it was 14.8 days [6]; and in China, it was 12 days [12]. This indicates that the length of hospitalization is different in different regions of the world when similar adult infections are compared; however, the number of studies comparing hospitalization length among different countries is too low to make an accurate comparison. Also, in this study, we included non-odontogenic infection, which may differ in terms of treatment progress of odontogenic infection. Finally, the length of hospital stay can be affected by financial factors. There is a difference in the cost of hospitalization because the system of health insurance is different in each country [16]. In the USA, daily mean room and bed charges ranged from $978 to $1598 [24]; on the other hand, in South Korea, they range from $30 to $200 per day [25] if the patient receives national health insurance. Due to expensive hospital costs, studies in the USA reported average hospital stay as shorter (3 to 8.3 days), compared with 12 days in this study.

The main limitation of this study was its retrospective study design. Because of the nature of retrospective studies, there is a need to rely on medical records to evaluate and measure the variables used in the study. Most studies regarding oromaxillofacial infections were conducted on a uniform group of patients, such as those with odontogenic or non-odontogenic infections. Moreover, previous studies assumed that the patient group and severity of symptoms in the environment of the emergency room are different from those of the outpatient setting. Hence, in future studies, it may be necessary to consider the differences between the emergency department patients and outpatients.

## Conclusions

From our findings, oromaxillofacial infection can be expected to heal well initially by removing the infection source and performing proper drainage, regardless of the factors associated with severity of infection. If the severity of the oromaxillofacial infection is assessed and an appropriately active surgical approach is initially undertaken, then the duration of the healing seems to be unaffected. The severity of the infection was not related to the duration of hospitalization and was found to be associated with factors affecting immunity such as patient age and concurrent diabetes. This study highlights the importance of adequate control of diabetes because patients with diabetes have a weakened immune system and control of infection is difficult.

In conclusion, this study identified that increasing age and the presence of concurrent diabetes are significant risk factors for a prolonged hospital stay during the treatment for oromaxillofacial infection.

## Abbreviations

CRP: C-reactive protein
I & D: Incision and drainage
